# Abnormal amplitude of low-frequency fluctuation values as a neuroimaging biomarker for major depressive disorder with suicidal attempts in adolescents: A resting-state fMRI and support vector machine analysis

**DOI:** 10.3389/fpsyg.2023.1146944

**Published:** 2023-02-24

**Authors:** Yang Zhou, Yu Song, Cheng Chen, Shu Yan, Mo Chen, Tao Liu

**Affiliations:** ^1^Department of Psychiatry, Wuhan Mental Health Center, Wuhan, Hubei, China; ^2^Department of Psychiatry, Wuhan Hospital for Psychotherapy, Wuhan, Hubei, China; ^3^Psychiatric Rehabilitation Department, Wuhan Mental Health Center, Wuhan, Hubei, China; ^4^Psychiatric Rehabilitation Department, Wuhan Hospital for Psychotherapy, Wuhan, Hubei, China; ^5^Department of Psychiatry, Suizhou Hospital, Hubei University of Medicine, Suizhou, Hubei, China

**Keywords:** amplitude of low-frequency fluctuation, major depressive disorder, suicidal attempts, resting-state fMRI, support vector machine

## Abstract

**Objective:**

Major depressive disorder (MDD) is associated with suicidal attempts (SAs) among adolescents, with suicide being the most common cause of mortality in this age group. This study explored the predictive utility of support vector machine (SVM)-based analyses of amplitude of low-frequency fluctuation (ALFF) results as a neuroimaging biomarker for aiding the diagnosis of MDD with SA in adolescents.

**Methods:**

Resting-state functional magnetic resonance imaging (rs-fMRI) analyses of 71 first-episode, drug-naive adolescent MDD patients with SA and 54 healthy control individuals were conducted. ALFF and SVM methods were used to analyze the imaging data.

**Results:**

Relative to healthy control individuals, adolescent MDD patients with a history of SAs showed reduced ALFF values in the bilateral medial superior frontal gyrus (mSFG) and bilateral precuneus. These lower ALFF values were also negatively correlated with child depression inventory (CDI) scores while reduced bilateral precuneus ALFF values were negatively correlated with Suicidal Ideation Questionnaire Junior (SIQ-JR) scores. SVM analyses showed that reduced ALFF values in the bilateral mSFG and bilateral precuneus had diagnostic accuracy levels of 76.8% (96/125) and 82.4% (103/125), respectively.

**Conclusion:**

Adolescent MDD patients with a history of SA exhibited abnormal ALFF. The identified abnormalities in specific brain regions may be involved in the pathogenesis of this condition and may help identify at-risk adolescents. Specifically, reductions in the ALFF in the bilateral mSFG and bilateral precuneus may be indicative of MDD and SA in adolescent patients.

## 1. Introduction

Major depressive disorder (MDD) is an extremely debilitating neuropsychiatric disease that causes characteristic and often severe emotional dysregulation (Wu et al., 2020). MDD is strongly associated with age and adolescents in the United States exhibit lifetime and 12-month MDD prevalence rates of 11 and 7.5%, respectively (Avenevoli et al., 2015). Adolescents suffering from MDD are also at risk of a range of other comorbid psychiatric conditions, suicide attempts (SAs; Doruk Camsari et al., 2019), substance abuse, and reduced social skills (Nardi et al., 2013); thus, the disease is a major focus of clinical psychiatry and public health-focused research throughout the world (Chen et al., 2020). MDD is the most common disorder present in adolescents that commit or attempt suicide (Nock et al., 2013), with suicide remaining the second most common driver of mortality among individuals 10–19 years of age (Breslin et al., 2020). Despite the severe toll that adolescent MDD can have on patients and those around them, the neurophysiological basis for this condition is not completely understood.

A range of magnetic resonance imaging (MRI) strategies have been used to assess patients with MDD. These include functional MRI (fMRI), magnetic resonance spectroscopy, structural MRI, and diffusion tensor imaging (Gao et al., 2021). Of these, fMRI offers value as a safe, noninvasive, reproducible tool that can aid in diagnosing disease through the assessment of minute shifts in blood oxygenation-level-dependent (BLOD) MRI signaling linked to brain activity (Gore, 2003). fMRI includes both resting-state (rs-fMRI) and task-based formats (Gao et al., 2021), with rs-fMRI performed in a quiet setting while subjects have their eyes closed and are not performing any tasks. Research by Biswal et al. suggests that the spontaneous resting-state BLOD signals observed under these conditions are reflective of basal neuronal activity (Biswal et al., 1995). Accordingly, rs-fMRI offers value as a means of examining spontaneous brain function and detecting any abnormalities or aberrant functional connectivity within the central nervous system (Biswal et al., 1995; Biswal, 2012). Unlike task-based fMRI analyses, rs-fMRI permits the evaluation of individuals not performing complex tasks and is thus better suited to the assessment of individuals unable to complete cognitively demanding tasks due to neurological or psychiatric disorders (Takamura and Hanakawa, 2017). For these reasons, fMRI had been used to study patients with a range of diseases and disorders, including depression (Guo et al., 2022), bipolar disorder (Vargas et al., 2013), schizophrenia (Whitfield-Gabrieli et al., 2009), epilepsy (Gao et al., 2022b), attention deficit hyperactivity disorder, abnormal brain development (Jolles et al., 2011), migraine (Wang et al., 2022), mild cognitive impairment (Gao et al., 2022c), and Parkinson’s Disease (Mi et al., 2021).

The two parameters that are most commonly calculated from BLOD signals recorded during rs-fMRI analyses include the amplitude of low-frequency fluctuation (ALFF) and functional connectivity (FC) (Zhang et al., 2014). Of these, ALFF is an indicator of the intensity of spontaneous local neuronal activity under these basal conditions. Shifts in spontaneous brain activity are assessed by fast Fourier transform of time-series data into the frequency domain and then examining the average amplitude from 0.01 to 0.08 Hz to compare changes in BOLD signals (Zang et al., 2007). Unlike FC, ALFF is a frequency-specific signal that is related to oscillatory phenomena, and directly reflects the intensity of spontaneous neural activity in a given region of the brain. While some studies have explored ALFF values in MDD patients, there is limited analysis of the results using support vector machine (SVM) techniques. SVM machine learning algorithms classify high-dimensional data points through the maximization of margins between classes (Pereira et al., 2009). SVM methods are frequently employed in psychiatric and neurological settings due to their high degree of classification accuracy and ability to process high-dimensional data (Gaonkar et al., 2015; Li et al., 2021).

Here, an SVM approach was employed to identify brain regions showing differences in ALFF values between adolescent MDD patients with a history of SA and healthy control individuals. The relationships between these values and patient depression and suicide scale scores were also assessed, and the utility of these changes in ALFF values as a neuroimaging biomarker of MDD with SA in adolescents was examined.

## 2. Materials and methods

### 2.1. Participants

A total of 71 first-episode drug-naïve adolescent depression patients were recruited from the Department of Psychiatry of Wuhan Mental Health Center. Patients were diagnosed with depression by two experienced psychiatrists based on DSM-IV criteria. To be eligible for study inclusion, patients had to be 7–17 years of age, right-handed, meet the diagnostic criteria for an acute episode of depression, have a history of SA within the past 14 days, be free of serious physical illnesses, be free of the alcohol and/or substance abuse or dependence, and be free of other Axis I disorders including schizophrenia, bipolar disorder, and substance-induced mood disorders. In addition, 54 age- and sex-matched healthy control individuals were recruited from the Wuhan Mental Health Center medical examination center. These controls were right-handed, had no history or family history of psychiatric disorders, and were free of any severe physical illness. All participants provided written informed consent for study participation. The Ethics Committee of Wuhan Mental Health Center approved this research, which was conducted in accordance with the guidelines of the Declaration of Helsinki.

SA was defined as any self-destructive behavior intended to terminate one’s own life that did not result in death (O’Carroll et al., 1996; Li et al., 2021). The patients included in this study were confirmed to have a history of SA through interviews with experienced psychiatrists, who also collected relevant details including the numbers of SAs and the dates on which they had occurred. When ambiguous results were obtained, the psychiatrists also made inquiries with the parents or clinicians of that patient to confirm these results. The Suicidal Ideation Questionnaire Junior (SIQ-JR; Keane et al., 1996) scale was conducted on the same day as the rs-fMRI to evaluate the severity of suicidal ideation, while the child depression inventory (CDI; Akimana et al., 2019) was used to assess depression severity.

### 2.2. Image acquisition

An Achieva 3 T MRI scanner (Philips, The Netherlands) was used for all imaging acquisition. The rs-fMRI data were preprocessed using MATLAB DPARSF software (Wang et al., 2017), as previously described (Gao et al., 2022a). Further details are provided in the Supplementary material.

### 2.3. ALFF analysis

ALFF analyses were performed with Rest software^1^ as reported previously (Zou et al., 2008). Further details are provided in the Supplementary material.

### 2.4. Classification analysis

SVM methods were used to evaluate the ability of ALFF values in specific brain regions to differentiate between MDD patients with a history of SA and healthy control individuals. The SVM analysis was conducted using the LIBSVM package in MATLAB; further information is provided in the Supplementary material.

### 2.5. Statistical analysis

Age, CDI scores, and years of education were compared between MDD patients and control individuals using two-sample *t*-tests, whereas gender distributions were compared with Chi-square tests. SPSS 22.0 was used for statistical analyses. Correlations between abnormal ALFF values and specific clinical findings were assessed *via* Pearson correlation analyses. *p* < 0.05 were considered significant.

A voxel-by-voxel covariance analysis of individual whole-brain ALFF maps was used to detect differences between the two study cohorts. Analyzed covariates included age, years of education, and framewise displacement. REST was used for the GRF correction of results at *p* < 0.01 (cluster significance: *p* < 0.01, voxel significance: *p* < 0.001).

## 3. Results

### 3.1. Participant characteristics

A total of 71 first-episode MDD patients with a history of recent SAs were enrolled in the study, together with 54 healthy control individuals were enrolled. The clinical and demographic characteristics of the participants are shown in Table 1. No significant differences in age, sex, or education level were observed between the groups.

**Table 1:** Characteristics of the participants.

Characteristics	Patients (*n* = 71)	HCs (*n* = 54)	*p* value
Gender (male/female)	71 (39/32)	54 (24/30)	0.245
Age, years	13.97 ± 1.51	14.17 ± 1.48	0.472
Years of education, years	6.79 ± 2.16	7.24 ± 2.28	0.261
CDI	30.27 ± 7.68	7.94 ± 2.64	0.000
SIQ-JR	65.58 ± 9.44	-	-

The value of *p* for gender distribution was obtained by the Chi-square test. The *p*-values were obtained by two sample *t*-tests. HC, healthy controls; CDI, child depression inventory; SIQ-JR, Suicidal Ideation Questionnaire Junior.

### 3.2. ALFF differences between groups

**Figure 1:**
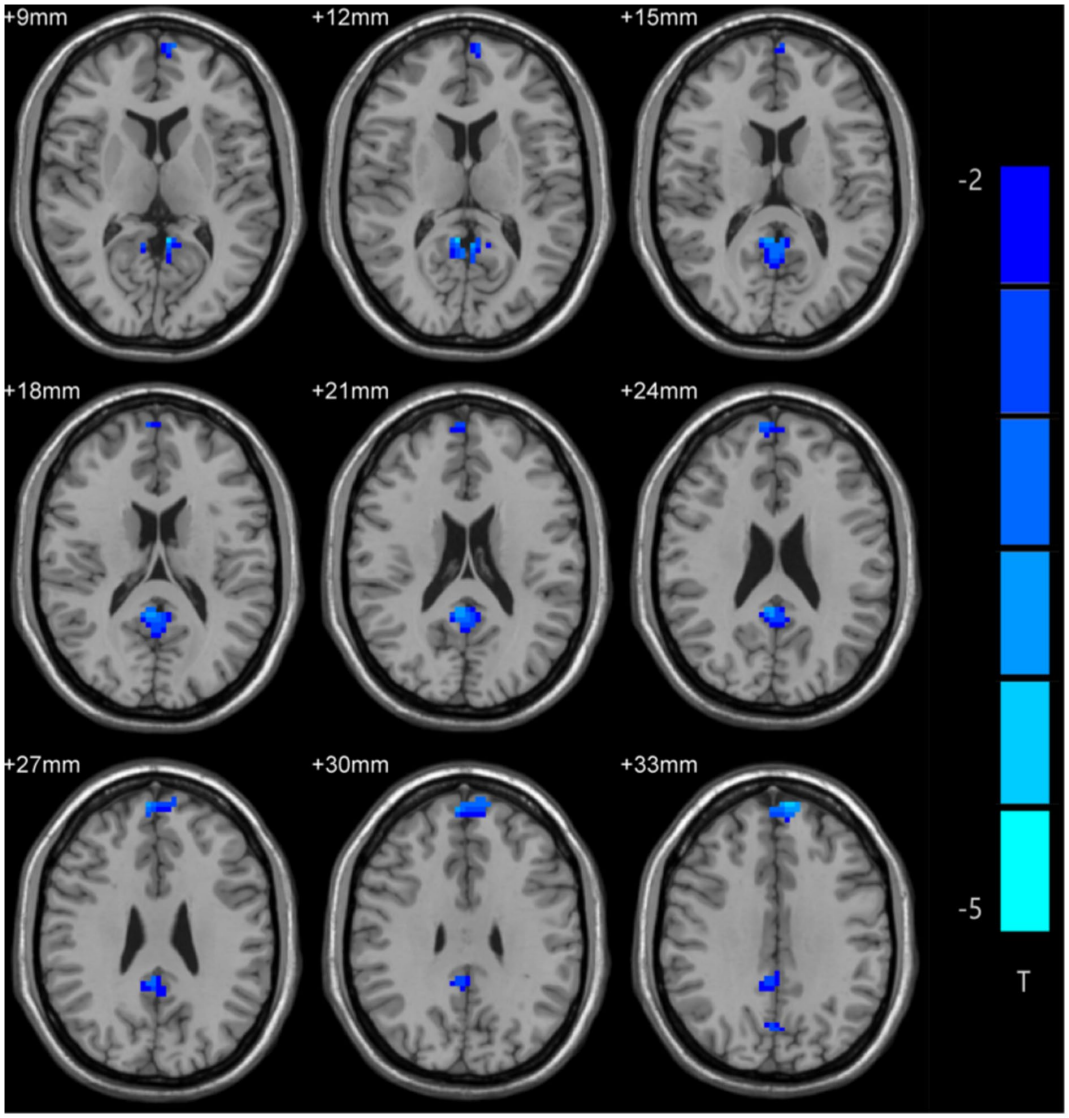
Amplitude of low-frequency fluctuation (ALFF) differences across groups. Blue color denotes low ALFF, and the darker the color, the lower the ALFF value of the brain area.

Differences in ALFF values were compared between MDD patients and controls using two-sample t-tests, revealing significantly lower ALFF values in the bilateral medial superior frontal gyrus (mSFG) and bilateral precuneus of MDD patients with a history of SA compared with the controls (Figure 1; Table 2).

**Table 2:** Signification differences in ALFF values between the groups.

Cluster location	Peak (MNI)			Number of voxels	*T* value
	X	Y	Z		
Bilateral mSFG	±9	57	39	103	−5.79
Bilateral precuneus	±3	−66	39	253	−4.97

ALFF, amplitude of low-frequency fluctuation; mSFG, medial superior frontal gyrus; MNI, Montreal Neurological Institute.

### 3.3. SVM results

**Figure 2:**
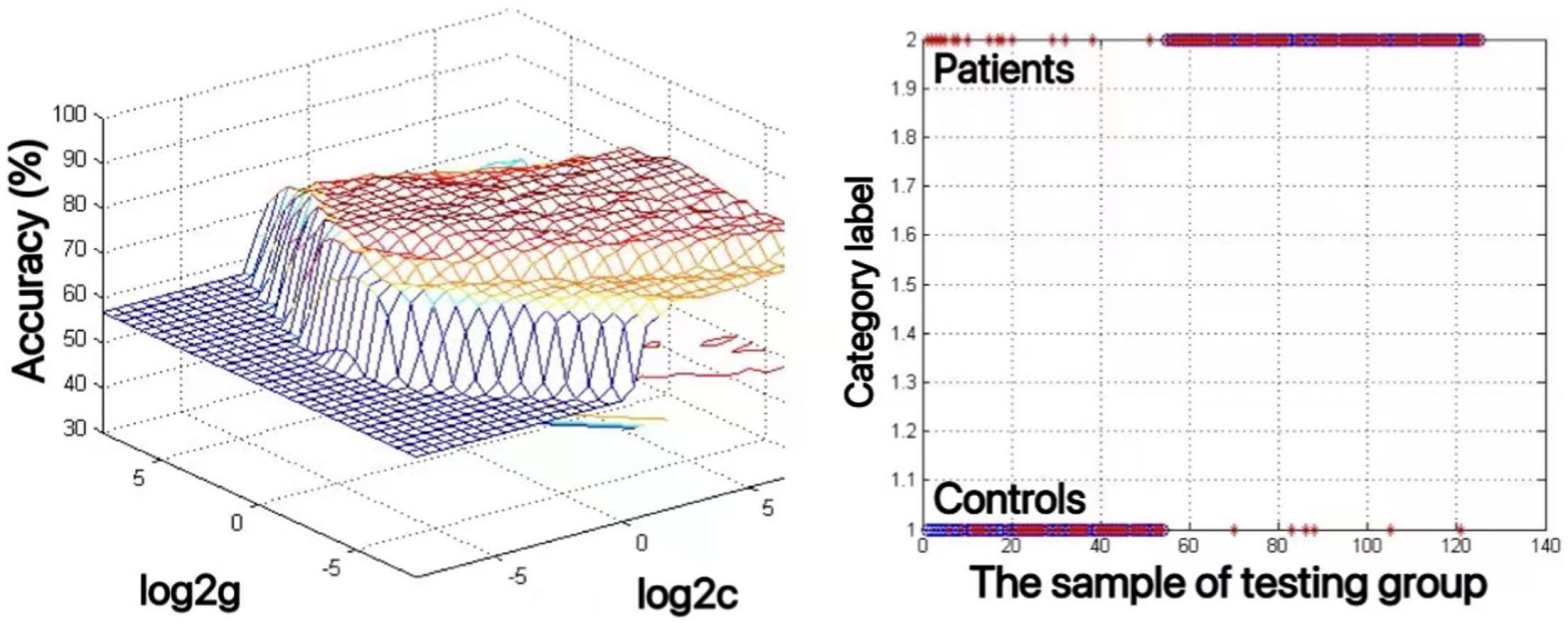
The use of decreased ALFF values in the bilateral precuneus to differentiate adolescents with major depressive disorder with suicidal attempts from healthy controls. Visualization of classifications through support vector machine (SVM) techniques using the ALFF values in the bilateral precuneus. Left: SVM parameters selection result of 3D view; Right: Classification map of the ALFF values in the bilateral precuneus.

An SVM approach was used to analyze decreases in the ALFF values of the bilateral mSFG and bilateral precuneus in MDD patients with a history of SA. This showed that reduced ALFF values in the bilateral precuneus offered the highest diagnostic accuracy of 82.4% (103/125), with a sensitivity and specificity of 91.5% (65/71) and 70.4% (38/54), respectively (Figure 2). The accuracy value for the reduced ALFF values in the bilateral mSFG was 76.8% (96/125) (data not shown).

### 3.4. Correlation results

**Figure 3:**
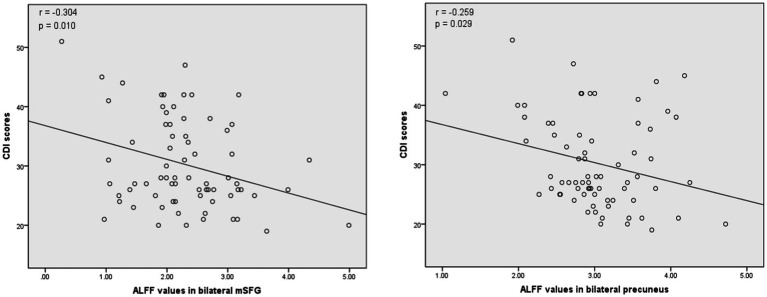
Correlations between abnormal ALFF and child depression inventory (CDI) scores. Left: Negative correlation between the ALFF values in the bilateral medial superior frontal gyrus (mSFG) and CDI scores. Right: Negative correlation between the ALFF values in the bilateral precuneus and CDI scores.

**Figure 4:**
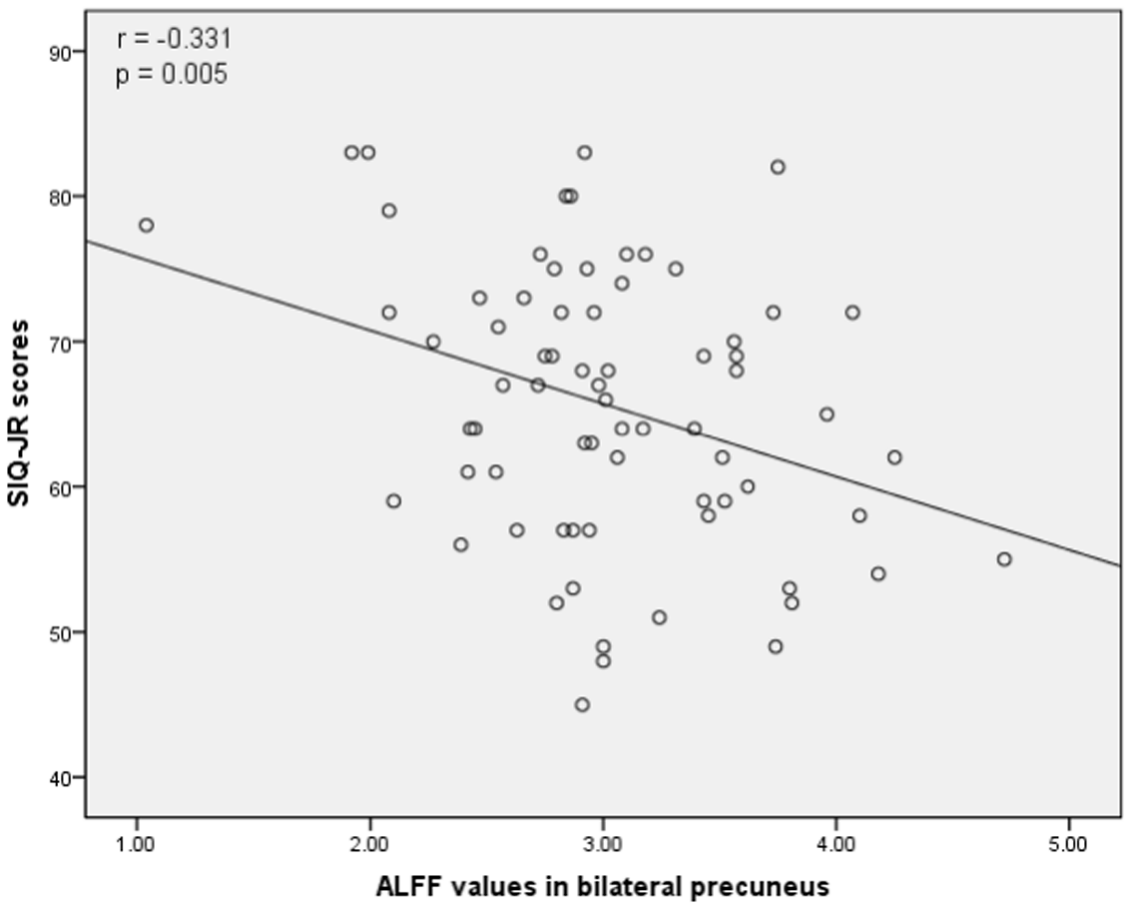
Correlations between abnormal ALFF and Suicidal Ideation Questionnaire Junior (SIQ-JR) scores. A negative correlation was found between the ALFF values in the bilateral precuneus and SIQ-JR scores.

The associations between ALFF values and other clinical variables in MDD patients were assessed through Pearson correlation analyses. This revealed the ALFF values in the bilateral mSFG and bilateral precuneus were negatively correlated with CDI scores (Figure 3), while there was also a negative correlation between ALFF values in the bilateral precuneus and SIQ-JR scores (Figure 4). ALFF values were not significantly correlated with patient age or years of education (data not shown).

## 4. Discussion

This is the first report of the use of an SVM technique to detect alterations in rs-fMRI-derived ALFF values in adolescent MDD patients with a history of SA compared with healthy controls. Significant reductions in the ALFF values were found in the mSFG and precuneus of MDD patients compared with the controls. Further analysis using SVM methods confirmed that these neuroimaging alterations may be of diagnostic value for the identification of adolescents with MDD.

The prefrontal cortex is positioned anteriorly to the premotor and motor portions of the frontal lobe (Lewis, 2004) and includes the mSFG, orbital SFG, and dorsolateral SFG (Liu et al., 2021). The default mode network (DMN) consists of an interconnected series of regions in the brain that exhibits higher levels of activity under resting conditions than during task completion. The mSFG is a DMN hub (Franco et al., 2009) and has been reported to be associated with aberrant neurological activity in patients with MDD (Guo et al., 2018). The mSFG also serves as an essential region of the brain necessary for emotional processing, executive function, the detection of causality, and for sequence learning (Van Overwalle, 2009). Previous studies have explored the association between the mSFG and MDD, with Peng et al., for example, reporting increased mSFG gray matter volume in these patients (Peng et al., 2016) while Liu et al. reported an association between dysfunction of the right mSFG and the cognitive processing of negative emotion in MDD (Liu et al., 2021). Here, ALFF values in the bilateral mSFG were found to be lower in MDD patients. In line with these results, a case–control rs-fMRI study of first-episode drug-naïve adolescent MDD patients showed lower ALFF values in the left medial frontal lobe (Gong et al., 2014). However, one voxel-based meta-analysis found higher ALFF values in the SFG in MDD patients than in controls (Gong et al., 2020). These differences may be linked to variations in study cohorts, duration of illness, medication use, and scanning parameters. For example, while higher bilateral SFG ALFF values were reported in early-onset depression, reduced values were found in late-onset depression (Guo et al., 2013). Another analysis that focused on non-suicidal self-harm also observed significantly lower bilateral mSFG ALFF values in adolescents suffering from MDD (Huang et al., 2021). Moreover, lower ALFF values in the right ventral medial frontal gyrus were reported in MDD patients with a history of SA compared with non-suidical patients (Fan et al., 2013). This may suggest a close relationship between ALFF values in the frontal gyrus and the risk of self-harm or suicide in MDD. Here, the SVM results indicated a 76.8% accuracy in the use of the these values to differentiate between adolescent patients with MDD and a history of SA and healthy controls. Correlation analysis showed that the mSFG ALFF values were negatively correlated with the and CDI scores but were unrelated to the SIQ-JR scores. Changes in the ALFF values in the mSFG may thus be closely related to the pathophysiology of MDD and/or suicidal ideation in these patients.

The precuneus has a unique anatomical location within the posteromedial parietal cortex buried in the interhemispheric fissure. It is thus rarely injured in isolation and it is particularly challenging to study (Cavanna and Trimble, 2006). The functions of the precuneus include self-awareness, cognition, autobiographical memory, and visuospatial processing (Zhang and Li, 2012; Tanglay et al., 2021). Much like the mSFG, the precuneus functions as a core DMN hub, and prior work suggests a close relationship with MDD. For example, in one rs-fMRI study, ALFF values in the left precuneus were found to be abnormal in individuals affected by social anxiety disorders and to be negatively correlated with the clinical symptoms experienced by these patients (Yuan et al., 2018). Lower ALFF values in the right precuneus have also been reported in MDD, which were found to remain below those of healthy controls even after remission, suggesting that ALFF values may represent a valuable biomarker for MDD (Wang et al., 2020). Consistently, other studies have proposed the application of ALFF as a diagnostic neuroimaging biomarker of MDD in both adolescent and adult populations (Gong et al., 2014; Li et al., 2018; Gong et al., 2020). Patients with depression accompanied by suicidal tendencies also exhibit reductions in right precuneus FC compared with controls (Shu et al., 2022). Consistent with these findings, the present study found that adolescent MDD patients with a history of SA showed lower ALFF values in the precuneus relative to healthy controls, and negative correlations were observed between the precuneus ALFF values and both CDI and SIQ-JR scores. The accuracy of the SVM results was 82.4%, indicating that the precuneus may represent a key mediator of MDD and SA pathogenesis. A reduced precuneus ALFF value should thus be explored as a neuroimaging biomarker for the diagnosis of MDD with suicidal tendencies in adolescents.

## 5. Summary and conclusion

MDD and suicidality represent growing public health problems throughout the world, especially among adolescents. Despite this pressing issue, the current understanding of MDD in adolescents is less understood compared with adults. The advent of novel neuroimaging technologies has led to the establishment of rs-fMRI as a valuable noninvasive means of aiding patient diagnosis in clinical settings. ALFF values, in particular, enable researchers to detect abnormalities in spontaneous brain activity through the monitoring of brain energy metabolism (Jing et al., 2013). Here, an SVM algorithm, which is commonly used for data analysis in biomedical contexts, was employed, demonstrating the value of reduced ALFF values in the bilateral mSFG and bilateral precuneus as neuroimaging biomarkers capable of identifying adolescent MDD patients with a history of SA.

## Data availability statement

The raw data supporting the conclusions of this article will be made available by the authors, without undue reservation.

## Ethics statement

The studies involving human participants were reviewed and approved by Ethics Committee of Wuhan Mental Health Center. Written informed consent to participate in this study was provided by the participants’ legal guardian/next of kin.

## Author contributions

YZ: investigation, resources, data analysis, manuscript writing and submitting. YS: investigation, resources, software, manuscript writing. CC: investigation, data curtion, writing-review. SY: data curtion, writing-review and editing. MC: conceptualization, methodology. TL: project administration, supervision. All authors contributed to the article and approved the submitted version.

## Funding

The investigation was supported by the Students’ Mental Health Network Project (SMHN) and the grant from the Wuhan Municipal Health Commission (Grant No.WG17D02).

## Conflict of interest

The authors declare that the research was conducted in the absence of any commercial or financial relationships that could be construed as a potential conflict of interest.

## Publisher’s note

All claims expressed in this article are solely those of the authors and do not necessarily represent those of their affiliated organizations, or those of the publisher, the editors and the reviewers. Any product that may be evaluated in this article, or claim that may be made by its manufacturer, is not guaranteed or endorsed by the publisher.

## Footnotes

^1^ http://www.resting-fmri.sourceforge.net
