# Supplementary material for: Abnormal amplitude of low-frequency fluctuation values as a neuroimaging biomarker for major depressive disorder with suicidal attempts in adolescents: A resting-state fMRI and support vector machine analysis
Source: Front Psychol. 2023 Feb 24;14:1146944. doi: 10.3389/fpsyg.2023.1146944 (PMC9998935; doi:10.3389/fpsyg.2023.1146944)
Supplement: Supplementary file 1 [file Data_Sheet_1.docx]

Supplementary Material

Abnormal amplitude of low-frequency fluctuation as a potential imaging biomarker for adolescent major depressive depression with suicidal attempts: a resting-state fMRI study and support vector machine analysis

Yang Zhou^1,2†^, Yu Song^3,4†^, Cheng Chen^1,2†^, Shu Yan^1,2^, Mo Chen^1,2*^, Tao Liu^5*^

^1^Department of Psychiatry, Wuhan Mental Health Center, Wuhan, 430012, Hubei province, China;

^2^Department of Psychiatry, Wuhan Hospital for Psychotherapy ,Wuhan, 430012, Hubei province, China;

^3^Psychiatric Rehabilitation Department, Wuhan Mental Health Center, Wuhan, 430012, Hubei province, China;

^4^Psychiatric Rehabilitation Department, Wuhan Hospital for Psychotherapy,Wuhan, 430012, Hubei province, China;

^5^Department of Psychiatry, Suizhou Hospital, Hubei university of medicine, Suizhou, 441300, Hubei province, China.

^†^ These authors have contributed equally to this work and share first authorship

*** Correspondence:**

Tao Liu 1902105647@qq.com and Mo Chen chenmo066@hust.edu.cn* contributed equally to this work and share correspondence author

## Image Acquisition

Achieva 3TMRI scanner (Philips, The Netherlands) was used for functional magnetic resonance imaging (fMRI). Patients are asked to lie down and close their eyes, but remain awake. Cover your ears with foam padding and earplugs to reduce the effects of head movement and noise. Functional imaging has the following parameters: repetition time to echo time ratio (TR/TE) (2000/30ms), slice thickness (5mm), pitch (1mm), field of view (220×220mm) and flip angle (90◦) . Structural scanning (T1 weighted) parameters: spin echo sequence, repetition time (TR) = 20ms, echo time (TE) = 3.5ms, slice thickness = 1mm, field of view (FOV) = 220×220mm.

## Data Preprocessing

The rs fMRI imaging data were preprocessed using DPARSF software in MATLAB. The first 5 time points were removed. Slice time and head motion correction. No participant had a maximum displacement of more than 2 mm in x, y, or z, and a maximum rotation of more than 2°. Register each patient's structures into their functional images. The structures of each patient were divided and normalized according to the Montreal Neurological Institute (MNI) standard template, modulated spatial deformation and using 1 × 1 × 1. Finally, each patient's structure was also normalized to the MNI space using the transformation matrix function. During functional image normalization, head motion parameters, white matter signal, and CSF signal were used as removal covariates, and voxel size 3 × 3 × 3 mm3 was used as functional covariate. Subsequently, the acquired images were smoothed with a full width of 8 mm at half-maximal Gaussian kernel, band-pass filtered (0.01–0.1 Hz), and linearly debiased to reduce the effects of low-frequency drift and physiological high-frequency noise. Several spurious covariates were removed, including signals from central regions of white matter, 6 head motion parameters obtained by rigid body correction, and signals from ventricular ROIs. Global signal removal can introduce artifacts into the data and distort resting-state connectivity patterns. Therefore, we preserved the whole-brain signal.

## ALFF analysis

After Data Preprocessing, linear trend was removed. Then the fMRI data were temporally band-pass filtered (0.01 < f < 0.08 Hz) to reduce the very low-frequency drift and high frequency respiratory and cardiac noise. ALFF analysis was performed using the REST software. The time series for each voxel was transformed to the frequency domain and the power spectrum was then obtained. Since the power of a given frequency is proportional to the square of the amplitude of this frequency component, the square root was calculated at each frequency of the power spectrum and the averaged square root was obtained across 0.01–0.08 Hz at each voxel. This averaged square root was taken as the ALFF. Spatial smoothing was conducted on the Z maps with an isotropic Gaussian kernel of 8 mm of full-width at half-maximum. The Z maps were transformed to the Talairach and Tournoux coordinates and one-sided one-sample t-test was performed on the Z maps.

## Classification Analysis

We use the LibSVM approach, which is a library about SVM developed by Professor Lin et al. in 2001. It is a flexible small program with few input parameters, open source, and easy to extend, so it has become the most widely used SVM library. This library tool can be accessed at https://www.csie.ntu.edu.tw/~cjlin/. In this experiment, the radial basis function (RBF) is used as the kernel function, which is also the default setting in LibSVM. Before using Weka to build a classification model, two parameters need to be determined, namely cost (c) and gamma (g). The parameter c is called the penalty coefficient. The higher the value of c, the easier it is to overfit. And g is a parameter after the RBF function is selected as the kernel, which affects the speed of the training and prediction process. There is no recognized best method for parameter selection. The common method is to let c and g take values within a certain range, and then set different c and g in the process of classifying the training set data. Finally, cross-validation is used to obtain the classification accuracy of the training set validation in this group c and g, and the group with the best classification result is selected by comparison. This is a complicated process, but in the LibSVM toolkit, parameter optimization is automated and manual tuning is no longer required. We use the program grid.py in the LibSVM tools folder to obtain the optimal parameters.
